# Development of evidence-based clinical practice guidelines (CPGs): comparing approaches

**DOI:** 10.1186/1748-5908-3-45

**Published:** 2008-10-27

**Authors:** Tari Turner, Marie Misso, Claire Harris, Sally Green

**Affiliations:** 1 Centre for Clinical Effectiveness, Southern Health, Locked Bag 29, Clayton, Victoria, 3168, Australia; 2 Monash Institute of Health Services Research, Monash University, Locked Bag 29, Clayton, Victoria, 3168, Australia; 3 Australasian Cochrane Centre, Monash Institute of Health Services Research, Monash University, Locked Bag 29, Clayton, Victoria, 3168, Australia

## Abstract

**Background:**

While the potential of clinical practice guidelines (CPGs) to support implementation of evidence has been demonstrated, it is not currently being achieved. CPGs are both poorly developed and ineffectively implemented. To improve clinical practice and health outcomes, both well-developed CPGs and effective methods of CPG implementation are needed. We sought to establish whether there is agreement on the fundamental characteristics of an evidence-based CPG development process and to explore whether the level of guidance provided in CPG development handbooks is sufficient for people using these handbooks to be able to apply it.

**Methods:**

CPG development handbooks were identified through a broad search of published and grey literature. Documents published in English produced by national or international organisations purporting to support development of evidence-based CPGs were included. A list of 14 key elements of a CPG development process was developed. Two authors read each handbook. For each handbook a judgement was made as to how it addressed each element; assigned as: 'mentioned and clear guidance provided', 'mentioned but limited practical detail provided ', or 'not mentioned'.

**Results:**

Six CPG development handbooks were included. These were produced by the Council of Europe, the National Health and Medical Research Council of Australia, the National Institute for Health and Clinical Excellence in the UK, the New Zealand Guidelines Group, the Scottish Intercollegiate Guideline Network, and the World Health Organization (WHO).

There was strong concordance between the handbooks on the key elements of an evidence-based CPG development process. All six of the handbooks require and provide guidance on establishment of a multidisciplinary guideline development group, involvement of consumers, identification of clinical questions or problems, systematic searches for and appraisal of research evidence, a process for drafting recommendations, consultation with others beyond the guideline development group, and ongoing review and updating of the CPG.

**Conclusion:**

The key elements of an evidence-based CPG development process are addressed with strong concordance by existing CPG development handbooks. Further research is required to determine why these key elements are often not addressed by CPG developers.

## Background

Clinicians want to give their patients the best possible care. To do this, they need to keep up to date with the evolving body of scientific research, and combine this scientific knowledge with their own clinical experience and each individual patient's circumstances and preferences. This is evidence-based practice [1].

While there is a growing acceptance of the importance of taking an evidence-based approach to clinical decision-making, in many areas across the spectrums of speciality and location, clinical practice is still not evidence-based [2]. Given the enormous potential health benefits of doing what we know works, it is essential that we find ways of implementing evidence into practice.

One way of supporting implementation of evidence is through evidence-based clinical practice guidelines (CPGs) [3,4]. CPGs outline a plan of expected care, providing a guide to recommended practice and outlining the likely outcomes of care [5]. They provide a guide to best practice, a framework within which clinical decisions can be made, and are used as a benchmark against which clinical practice can be evaluated. Historically, CPGs were often developed by consensus of a group of expert clinicians without explicit reference to research evidence. Evidence-based CPG development emphasises the importance of linking recommendations to the scientific research that supports them, identified through a rigorous systematic identification and appraisal of all relevant research.

While the potential of CPGs to support implementation of evidence has been demonstrated, it is not currently being achieved [6]. To improve clinical practice and health outcomes, both well-developed CPGs and effective methods of CPG implementation are needed. Currently, there are weaknesses to be addressed in to both these aspects.

CPGs are increasingly being developed at local, regional, and national levels [4,7]. The increase in publications about the CPG field gives an indication of the rapid rise of CPG development. In 1993, the medical subject heading (MeSH) 'practice guideline' was added to MEDLINE, and 444 articles were classified under that heading. In the 13 subsequent years, there has been a more than eleven-fold increase in the number of articles classified per year under the 'practice guideline' MeSH, to a total of 4,975 articles in 2006. In comparison, over the same period of time, the number of articles per year indexed in Medline has not quite doubled, increasing from 413,375 in 1993 to 727,546 articles in 2006.

In parallel with the increasing interest in CPGs, several groups responsible for producing evidence-based CPGs have published handbooks outlining the development methodology [8-13]. These development handbooks are substantial bodies of work, describing in various levels of detail the methods by which evidence-based CPGs should be developed. These handbooks provide guidance to CPG developers working within the authoring organisations, and therefore influence the quality of CPGs produced by these groups. However, in addition, the handbooks are used much more widely by CPG developers in other organisations, such as professional colleges, peak bodies for clinical conditions, health services, and government departments. As a result, the CPG development methods proposed in these handbooks have the potential to influence the quality of hundreds, if not thousands of CPGs. At the same time, the AGREE (Appraisal of Guidelines Research and Evaluation) Collaboration has produced an instrument which is widely used to assess the validity of CPGs and which includes several criteria addressing the rigour of development of the CPG [14].

However, in spite of the massive increase of interest in CPGs, and the availability of CPG development handbooks, several studies in a number of countries including Australia, Canada, the United Kingdom, and Finland have demonstrated that few CPGs meet quality criteria, such as those outlined by AGREE, and that the link between research and recommendations is an area of particular weakness [15-24]. This mismatch between recommended and actual CPG development processes has also recently been highlighted in a review of use of evidence in World Health Organisation guidelines [25]. The reasons for this gap are unclear.

If the available handbooks provide differing guidance, then perhaps it is reasonable that CPG developers are unclear as to the components of a CPG development process, and this might explain some of the weaknesses observed in CPG development. If this is the case, it is imperative to ascertain what the differences in guidance are, whether these differences are gaps or contradictions in guidance, and, importantly, why these differences occur. On the other hand, if all the handbooks provide consistent advice, then perhaps this duplication represents wasted effort, and one handbook that synthesises the best of each and is regularly updated, supplemented by additional materials to meet the needs of each particular organisation, would be a better use of resources.

As part of a wider study investigating methods of evidence-based CPG development in hospital settings, we sought to compare the advice of CPG development handbooks produced by major national or international organisations that have a role in developing standards for evidence-based CPGs. We aimed to establish whether there was agreement on the fundamental characteristics of an evidence-based CPG development process. Further, we were interested to explore whether the level of guidance provided in these handbooks was sufficient for people using these handbooks to be able to apply it and develop evidence-based CPGs.

## Methods

### Search strategy

We identified CPG development handbooks through a broad search of MEDLINE using search terms including 'guideline* and evidence and (method* or develop*)'. We searched CPG websites such as the US National Guidelines Clearinghouse  and the Guidelines International Network , websites of known CPG development bodies, and the internet using Google. We also asked members of the evidence-based health care email list "evidence-based-health@jiscmail.ac.uk" to suggest potentially relevant documents.

### Inclusion criteria

We included documents published in English produced by national or international organisations responsible for CPG development. We only included those documents purporting to support development of evidence-based CPGs, not consensus-only or non-evidence-based CPGs. We excluded documents produced by professional colleges or peak bodies for specific conditions, as we were aiming to identify documents that were used widely beyond the organisation in which they were developed.

### Data extraction

We initially planned to use the AGREE instrument [14] to compare the advice provided in the handbooks. However, the AGREE instrument does not include criteria to address several areas of evidence-based CPG development, such as the appraisal of research studies and formation of an implementation plan. To identify other key elements not included in AGREE, we reviewed the wider guideline development literature and drew from literature including the Cluzeau instrument [26] from which AGREE was derived, and other authors reporting on the components of an evidence-based CPG development process [27,28] to develop a more comprehensive list. On the basis of the literature, one author (TT) developed a draft list of key elements of the CPG development process. These elements were reviewed by two other authors (CH, SG) and consensus was reached through discussion. As a result, we developed a list that included the key elements of a development process addressed by AGREE, but which also included additional elements.

To establish face validity, a draft list of evidence-based CPG development elements was circulated to the evidence-based health care email list, and to leaders in evidence-based CPG methods for their comments. As a result, the list was reordered and the wording of some elements was refined to reduce opportunity for misinterpretation.

Fourteen key elements of the development process were identified (Table 1). These key elements relate broadly to four phases of CPG development: preparing for development, systematically reviewing the evidence, writing the CPG, and reviewing the CPG. However, they should not be interpreted as reflecting a strictly linear process. Developing an implementation strategy, for example, may begin very early in the CPG development process. The list of elements was neither exhaustive nor proscriptive. It was designed to include the major elements of an evidence-based CPG development process; it was not designed as a checklist of what should be in a handbook, but as a framework which would allow comparison of what was in each handbook.

**Table 1:** Key elements of CPG development

**Key Element**	**Phase of Development**
• Selecting topic	Preparing for CPG development
• Determining the scope of the CPG	
• Identifying and adapting existing CPGs	
• Forming a multidisciplinary guideline development group	
• Involving consumers	

• Establishing clinical questions	Systematically reviewing the evidence
• Systematic searching (including documentation of sources, filters and limits)	
• Including and/or excluding identified research	
• Appraising research	

• Developing recommendations	Drafting the CPG
• Developing an implementation strategy	
• Consulting on the draft CPG	
• Writing of summary versions of the CPG	

• Planning for evaluating the impact, revising and updating of the CPG	Reviewing the CPG

Two authors (TT, MM) independently read each handbook to identify whether it addressed each of the key elements. We were interested in whether the handbooks mentioned each element and, if so, whether they provided enough practical information for a CPG developer to understand not only what was required for each element of the process but also how to complete that element. For each key element, evidence was extracted in the form of direct quotations for illustration where possible, and a judgement of whether the handbook addressed each element then assigned of either 'not mentioned', 'mentioned but limited practical detail provided', or 'mentioned and clear practical guidance provided'. Disagreements were resolved through discussion. During the data extraction process, we also sought to identify any other key elements of the CPG development process that we had not included in our list 14 key elements. As no human participants were involved, ethics approval was not required.

## Results

Our search did not yield any handbooks of which we were not already aware. Six handbooks for developing CPGs were included. The six handbooks were produced by the Council of Europe, the National Health and Medical Research Council of Australia (NHMRC), the National Institute for Health and Clinical Excellence in the UK (NICE), the New Zealand Guidelines Group (NZGG), the Scottish Intercollegiate Guideline Network (SIGN), and the WHO. Table 2 details the extent to which each handbook addressed the key elements. No additional key elements in the CPG development process were identified during data extraction.

**Table 2:** Comparison of guidance provided by CPG development handbooks

Organisation	Council of Europe	NHMRC	NICE	NZGG	SIGN	WHO
Topic selection	✓✓	✓✓	Beyond scope	✓✓	✓✓	✓✓
Scoping the CPG	-	✓✓	✓✓	✓✓	✓	-
Adaptation of existing CPGs	✓	✓✓	✓	✓✓	✓	-
Multidisciplinary GDG	✓✓	✓✓	✓✓	✓✓	✓✓	✓✓
Consumer involvement	✓	✓✓	✓✓	✓✓	✓✓	✓
Clinical questions	✓	✓	✓✓	✓✓	✓	✓
Systematic searches	✓	✓	✓✓	✓✓	✓	✓
Inclusion and exclusion	-	✓	✓	✓	✓	-
Appraisal of evidence	✓	✓	✓✓	✓✓	✓✓	✓✓
Developing recommendations	✓	✓✓	✓✓	✓✓	✓✓	✓✓
Implementation plan	✓✓	✓✓	✓	✓✓	Beyond scope	✓
Consultation on draft	✓	✓✓	✓✓	✓	✓✓	✓
Writing summaries	-	✓	✓✓	✓✓	✓✓	-
Evaluation, revision and updates	✓✓	✓✓	✓✓	✓	✓✓	✓

✓✓ Mentioned and clear practical guidance provided; ✓ Mentioned, but limited practical detail;

- Not mentioned

Five of the six handbooks provide clear guidance on the way in which CPG topics are or should be selected. For example the SIGN handbooks states: *'*Guideline topics... are chosen on the basis of the burden of disease, the existence of variation in practice, and the potential to improve outcome.' The NICE handbook is the only handbook which does not address this aspect, the reason being that for NICE 'Guideline topics are selected by the Department of Health'.

Two of the handbooks (Council of Europe, WHO) make no mention of defining the scope of the CPG in terms of patient population, clinical condition, context or target audience. SIGN provides some mention and NICE, NZGG, and the NHMRC have very clear guidance and specific processes with the NHMRC stating: 'Before proceeding, the panel should clarify the purpose of and target audience for the guidelines. This will involve a careful specification of the following: the conditions and clinical problems that are at issue; the type of care providers for whom the guidelines are intended; the type of consumers for whom the guidelines are intended; a description of consumers not covered by the guidelines (where it might otherwise be assumed that the guidelines are relevant to these consumers); the types of settings in which the guidelines will be employed; and the interventions to be evaluated.'

Adaptation of existing CPGs is addressed in all except the WHO handbook. Varying levels of support for and details of the process are provided. The NZGG handbook states: 'If there is already a guideline in existence that addresses the same issue or problem, another guideline may not be needed. An evaluation and/or adaptation of that guideline may be more appropriate than developing a new guideline.' The NICE handbook differentiates between adaptation of NICE CPGs and adaptation of CPGs developed by other organisations. For NICE CPGs, the handbook suggests that the evidence summaries should be used in the form that they are found, and the wording of recommendations adapted as necessary. The handbook suggests that existing non-NICE CPGs can be used as evidence, but new evidence summaries should be created and new recommendations written.

The requirement for a multidisciplinary guideline development group is mentioned by all six handbooks, with all providing clear guidance, though the detail of the suggested makeup of the group varies, as does the emphasis on specific aspects of this process such as identification and management of conflicts of interest.

Consumer involvement is mentioned by all six handbooks. The degree to which consumer involvement is required, and the nature and level of detail in the advice on how this involvement should be facilitated vary widely. NHMRC, NICE, NZGG, and SIGN provide clear guidance, and WHO and The Council of Europe provide less detailed advice. The SIGN handbooks states: 'The involvement of patients, carers or their representatives in guideline development is therefore important to help ensure that guidelines reflect their needs and concerns.'

Formulation of clinical questions is only addressed in detail in the NICE, NZGG, and SIGN handbooks, though the other handbooks all mention the importance of defining the clinical problems to be addressed and provide limited guidance on how this should be done. While the NICE and NZGG handbooks provide useful guidance, for example, on how to format questions within a PICO (patient, intervention, comparison, outcome) framework, none of the handbooks address how to ascertain the clinical questions to be addressed by the CPG.

All six handbooks mention that systematic searches should be undertaken, and the NICE and NZGG handbooks provide clear guidance on how to do this, with practical details about which information sources to search and when to use filters or limits. Some handbooks seem to suggest that one systematic search may be enough for an entire CPG, others are clear that a systematic search should be undertaken for each clinical question addressed by the CPG. For example, the Council of Europe handbook states: 'At present, the preferred guideline development method is to search explicitly and systematically for pertinent evidence to answer each central question being addressed in the guideline.'

The NICE and SIGN handbooks provide clear guidance on processes for inclusion or exclusion of potentially relevant research evidence. The other handbooks provide more limited information.

All six handbooks address appraisal of evidence in some way, with NICE, NZGG and SIGN providing clear processes and templates.

Methods for drafting recommendations are at least mentioned by all six handbooks, with the Council of Europe providing more limited advice than the NHMRC, NICE, NZGG, WHO, and SIGN handbooks. NICE in particular provides detailed advice describing how to word recommendations including instructions that they should be 'standalone' and 'action-oriented'.

Similarly, all six handbooks mention the importance of facilitating consultation on the draft CPGs, for example, the WHO handbook states: 'Ensure peer review – Circulate widely to experts, professional organizations, regional offices, countries'. NICE, NHMRC, and SIGN have clear, well-defined consultation processes.

Writing of summaries is mentioned in all but the Council of Europe and WHO handbooks. The NZGG asserts that 'The majority of readers and users of CPGs will not have the time to read the whole document and it is essential that a summary of the guideline with recommendations is produced. Algorithms are also useful tools that can improve guideline utilisation.'

A process for developing an implementation strategy for inclusion in the CPG is addressed in all but the SIGN and NICE handbooks. Implementation is beyond the scope of the SIGN mandate, and the NICE handbook refers CPG developers to the NICE website for implementation materials. The Council of Europe, NHMRC, and NZGG handbooks provide particularly detailed practical guidance in this area.

Evaluation, review, and updating of CPGs is addressed by all of the handbooks, with the NHMRC pointedly stating: 'Evaluation of CPGs is essential. How will we know if guidelines are worth developing, disseminating and implementing if we do not know whether they make a difference to clinical practice and health outcomes?' The amount of practical detail available to enable guideline developers to carry out these processes varies between handbooks, and often discussion is quite limited.

## Discussion

This study has a number of limitations. Resource restrictions meant that we were only able to search for and include handbooks that were published in English. Our requirement that handbooks be developed by national or international organisations responsible for CPG development may mean that well-developed handbooks for guideline development were excluded, particularly in North America where several handbooks are produced by professional groups. Related resources such as the manual for the U.S. Preventive Services Task Force  were also excluded, as they did not meet our criteria, but may be relevant to this discussion. These restrictions were pragmatic, chosen *a priori*, and allowed application of objective criteria for inclusion, but we acknowledge that this is a limitation of our study. Another potential limitation of this study was the assessment of the degree to which a key element was addressed by each of the handbooks. By its nature, this type of assessment is subjective, and what constitutes sufficient practical detail to undertaken a particular step of the CPG development process will vary from one CPG author to another. In almost every case, however, the assessment of the handbooks by our two reviewers was very similar.

This review of six 'guidelines for developing guidelines' has highlighted a strong concordance on the central elements of an evidence-based CPG development process. All six of the handbooks require establishment of a multidisciplinary guideline development group, involvement of consumers, identification of clinical questions or problems, systematic searches for and appraisal of research evidence, a process for drafting recommendations, consultation with others beyond the guideline development group, and ongoing review and updating of the CPG. While the handbooks vary in the amount of detail they provide to CPG developers in these areas, fundamentally they agree that these are the key components of an acceptable CPG development process.

It is unlikely that these handbooks were developed independently of each other, though ironically, this is hard to assess as few of the handbooks explicitly detail the methods by which they were developed. For example the NHMRC, NICE, NZGG, and Council of Europe handbooks all mention the SIGN handbook, though none describe how it was used in their handbook development.

The degree of concordance between handbooks is interesting given the range of publication dates. The handbooks have been produced over an eight year period, with the earliest, the NHMRC 'Guide to the development, implementation and evaluation of CPGs' published in 1999, and the most recent, the NICE 'Guidelines Manual' released in April 2007. Eight years is a long time in any field, and in evidence-based practice a great deal has changed over the past eight years. The strong similarities between the processes recommended by these two handbooks, and the others published in the intervening period, may be viewed either positively, suggesting that the process of evidence-based CPG development is well established and widely accepted in CPG development organisations; or negatively, suggesting that development CPG processes are not progressing with changes in the field of evidence-based practice.

The concordance between handbooks on what constitutes evidence-based CPG development is particularly interesting given that research shows that many CPG developers are not following this approach [15-24]. If the advice is consistent then the mismatch between recommended and actual development processes may occur for a variety of reasons. Developers may be unaware of the recommended methods, handbooks may provide insufficient information on how to complete the steps of the process, developers may disagree with the recommended approach or the currently recommended process may be too complex, time-consuming, or resource-intensive to be achievable. Confusion resulting from the difference between the handbooks' description of the process, and the AGREE instrument assessment of the process, discussed further below, may also contribute to the problem. Further research could focus on identifying the barriers to evidence-based CPG development to establish if there is a need for more detailed advice, if the recommended methods are not feasible in some settings, or both.

While the handbooks provide information on the steps that make up the CPG development process, they often lack detail on how to carry out these steps in practice. A good example is the development of clinical questions. While all handbooks mention clinical questions and some provide a great deal of useful information on what constitutes a good clinical question, the PICO (patient, intervention, comparison, outcome) framework, the importance of ensuring clinical questions cover the whole scope of the guideline, etc., none of the handbooks provide advice on how to actually generate the clinical questions. This lack of practical detail is found in most aspects of the CPG development process and may explain some the variation seen in the quality of CPGs. On the basis of reading these handbooks, CPG developers may be aware of what is required in an evidence-based CPG development process but may not know how to do it. While detailed handbooks have been produced in related areas, such as the Cochrane Handbook of Systematic Reviews , and are sometimes referred to by CPG development handbooks, these have not been written with CPG developers in mind, and may not meet their specific needs.

Given the general agreement between handbooks and the increasing collaboration of CPG developers through organisations such as the Guidelines International Network, a question is also raised about the appropriateness of each of the CPG development organisations continuing to invest in updating, revising, and refining the content of these handbooks. This surely represents a duplication of effort. Perhaps the limited available resources might be better spent on a collection of shared materials which could be enriched in terms of practical detail, providing thorough guidance on how to complete each step in the process as well as the currently available guidance on 'what to do'. These shared materials could then be supplemented by smaller documents describing organisation specific processes or requirements.

This review also highlights some differences between the methods of evidence-based CPG development proposed by the handbooks, and the criteria used to assess the quality of CPGs. AGREE, a widely used method of CPG appraisal, does not include criteria addressing how the CPG topic was selected or how the CPG will be implemented. Perhaps more importantly, it also does not assess whether systematic methods were used to appraise the research evidence used to support CPG recommendations, which was considered a key step in a CPG development process by the handbooks reviewed in this study. The reason for these discrepancies is unclear, and they seem quite odd given that each of these three areas was addressed in the instrument from which AGREE was developed [26]. If a consistent approach to and standard of CPG development is to be advocated by both CPG developers and assessors, then these discrepancies need to be at the very least explained, and ideally, solved.

## Conclusion

There has been a rapid increase in the rates of development of CPGs. In the evidence-based CPG development handbooks reviewed in this study, there is strong concordance as to the key elements in this process: establishment of a multidisciplinary guideline development group, involvement of consumers, clear identification of clinical issues, systematic searches for and appraisal of research evidence, a process for drafting CPG recommendations and consultation with others beyond the GDG. While it seems that the key elements of an evidence-based CPG development process are well established, given that they are often not followed by developers, research is needed to determine the barriers to implementing the recommended CPG development methods; and perhaps more practical detail or a simpler approach to developing CPGs may be required.

## Competing interests

TT, CH, SG and MM have all been involved in evidence-based CPG development using methods adapted from the NHMRC, SIGN and NICE handbooks. MM and SG are staff of the Australasian Cochrane Centre, funded by the Australian Department of Health and Ageing and supported by Monash University. Sally Green is a member of the Cochrane Collaboration Steering Group. SG is an editor of the Cochrane Handbook of Systematic Reviews of Interventions which informs the methodology for Cochrane systematic reviews. The views expressed in this paper represent those of the authors and are not necessarily the views or the official policy of the Cochrane Collaboration (unless otherwise stated and referenced).

## Authors' contributions

TT conceived of this project and developed the methodology and data extraction approach, which TT, SG and CH refined. TT and MM undertook the data extraction. TT prepared the first draft of this article which CH, SG, MM and TT then revised. TT is guarantor for the content of this article.
